# Supplementary material for: Diversity and Complexity of Internally Deleted Viral Genomes in Influenza A Virus Subpopulations with Enhanced Interferon-Inducing Phenotypes
Source: Viruses. 2023 Oct 17;15(10):2107. doi: 10.3390/v15102107 (PMC10612045; doi:10.3390/v15102107)
Supplement: Supplementary file 1 [file viruses-15-02107-s001.zip › Supplementary Figures.pdf]

viruses

MDPI

Article

Diversity and Complexity of Internally Deleted Viral Genomes in Influenza A Virus Subpopulations with Enhanced Interferon-Inducing Phenotypes

Amir Ghorbani [1,2,†], John M. Ngunjiri [2,‡], Gloria Rendon [3], Christopher B. Brooke [3,4], Scott P. Kenney [1,2,*] and Chang-Won Lee [5,*]

[1] Department of Veterinary Preventive Medicine, College of Veterinary Medicine, The Ohio State University, Columbus, OH 43210, USA; amir.ghorbani@nih.gov
[2] Center for Food Animal Health, Ohio Agricultural Research and Development Center, The Ohio State University, Wooster, OH 44691, USA; john.ngunjiri@targan.com
[3] Carl R. Woese Institute for Genomic Biology, University of Illinois at Urbana-Champaign, Urbana, IL 61801, USA; gloriarendon@gmail.com (G.R.); cbrooke@illinois.edu (C.B.B.)
[4] Department of Microbiology, University of Illinois at Urbana-Champaign, Urbana, IL 61801, USA
[5] Southeast Poultry Research Laboratory, US National Poultry Research Center, USDA, ARS, Athens, GA 30605, USA
* Correspondence: kenney.157@osu.edu (S.P.K.); chang.lee@usda.gov (C.-W.L.)
† Current address: Cellular Biology Section, Laboratory of Viral Diseases, National Institute of Allergy and Infectious Diseases, National Institutes of Health, Bethesda, MD 20892, USA.
‡ Current address: TARGAN Inc., 350 E. Six Forks Rd., Raleigh, NC 27609, USA.

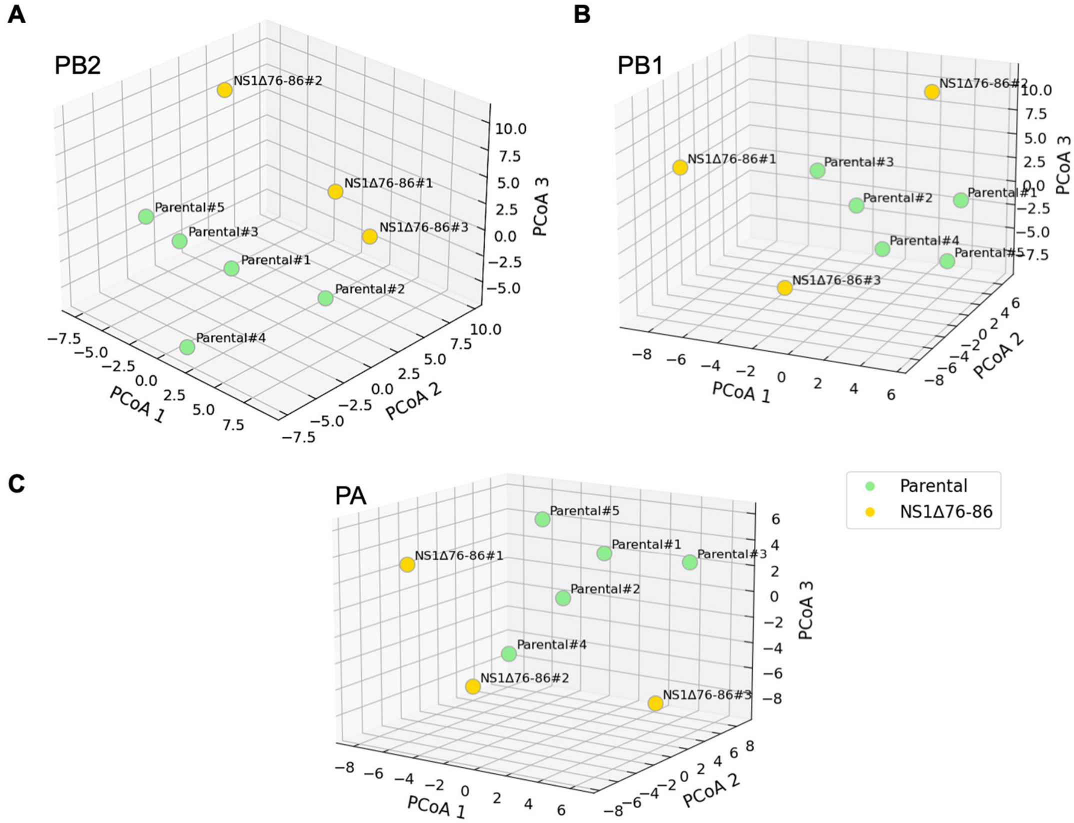

Supplementary Figure S1. Principal component analysis (PCoA) based on Euclidean distances among the abundance of ID vRNA and the break and rejoin sites for PB2 (**A**), PB1 (**B**), and PA (**C**)-derived ID vRNAs detected in parental (green) and NS1Δ76-86 (golden) clones. The NS1Δ76-86 clones appeared to have greater separation from each other than the parental clones.

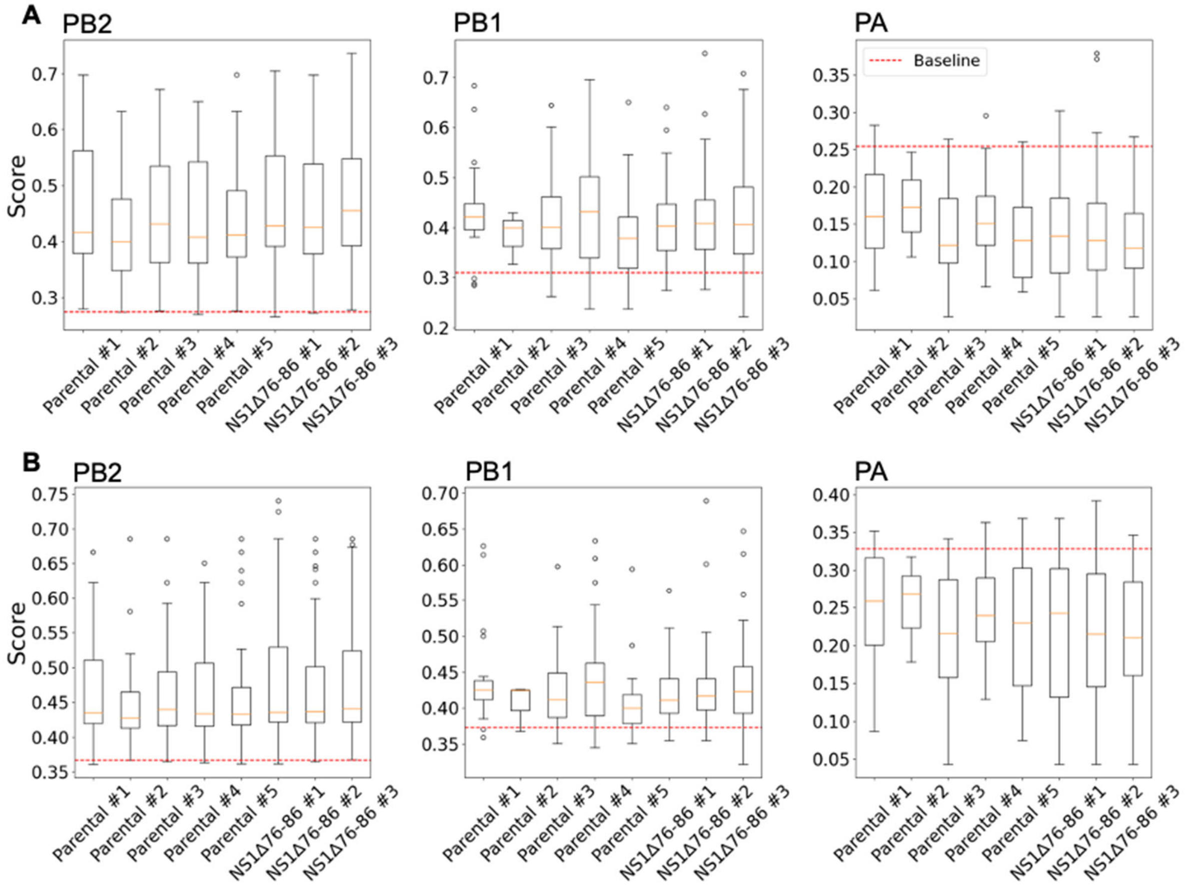

Supplementary Figure S2. Prediction of the disordered nature of putative viral polypeptides encoded by the ID vRNAs from each plaque-purified clone. No significant differences were observed between the average IUPred3 (**A**) and ANCHOR2 (**B**) values among the viral clones with distinct IFN-inducing phenotypes. Red dotted lines represent the mean value estimated for the intact PB2, PB1, or PA viral proteins.
